# Caputo SIR model for COVID-19 under optimized fractional order

**DOI:** 10.1186/s13662-021-03345-5

**Published:** 2021-03-24

**Authors:** Ali S. Alshomrani, Malik Z. Ullah, Dumitru Baleanu

**Affiliations:** 1 Department of Mathematics, King Abdul Aziz University, Jeddah, Saudi Arabia; 2 Department of Mathematics, Cankaya University, Öǧretmenler Cad. 1406530, Ankara, Turkey; 3 Institute of Space Sciences, Magurele, Bucharest, Romania; 4 Department of Medical Research, China Medical University Hospital, China Medical University, Taichung, Taiwan

**Keywords:** Ulam–Hyers stability, Least-squares, Caputo, Pandemic, SIR model

## Abstract

Everyone is talking about coronavirus from the last couple of months due to its exponential spread throughout the globe. Lives have become paralyzed, and as many as 180 countries have been so far affected with 928,287 (14 September 2020) deaths within a couple of months. Ironically, 29,185,779 are still active cases. Having seen such a drastic situation, a relatively simple epidemiological SIR model with Caputo derivative is suggested unlike more sophisticated models being proposed nowadays in the current literature. The major aim of the present research study is to look for possibilities and extents to which the SIR model fits the real data for the cases chosen from 1 April to 15 March 2020, Pakistan. To further analyze qualitative behavior of the Caputo SIR model, uniqueness conditions under the Banach contraction principle are discussed and stability analysis with basic reproduction number is investigated using Ulam–Hyers and its generalized version. The best parameters have been obtained via the nonlinear least-squares curve fitting technique. The infectious compartment of the Caputo SIR model fits the real data better than the classical version of the SIR model (Brauer et al. in Mathematical Models in Epidemiology 2019). Average absolute relative error under the Caputo operator is about 48% smaller than the one obtained in the classical case ($\nu =1$). Time series and 3D contour plots offer social distancing to be the most effective measure to control the epidemic.

## Introduction

Literature includes mathematical models for pathways for the transmission of infectious ailments. These models play an important role in quantifying and assessing the efficient control and preventive measures of infectious ailments [2–4]. It has been proved in multifarious ways that mathematical modeling is a very flexible and efficient way of researching the dynamics of transmission of infectious ailments. Mathematical analysis and numerical simulations can be used to create and evaluate control measures that are convincing. As for the model of compartmental ailments, there are several infectious ailments, beginning with the very classic SIR model to the more complex ones [1].

In December 2019, there was an emergence of a novel ailment in China which was later declared as pandemic named COVID-19 by the World Health Organization [5]. It is well known that the COVID-19 pandemic has given rise to fearful living and coexistence around the globe, with millions of people worldwide infected. The mortality-recovery ratio appears to be in a positive proportion. Nevertheless, due to the sensitivity of polymerase chain reaction, the absence or presence of the previously infected host is observed and the recovery rate appears to be promising in the absence of any curative vaccine. The challenge faced by health care professionals, the World Health Organization, and the Center for Disease Control in each quarter was whether reinfection could occur after a COVID-19 patient had been clinically treated. The subtle nature of the disease has led many scientists and medical practitioners to massively embark on multiple studies to combat and avoid the spread of the disease [6–11].

Out of the seven known human COVID-19, four are the known human influenza pathogens. SARS-CoV, MERS-CoV, and 2019-nCoV are responsible for severe respiratory diseases [12]. While COVID-19 has long been developed and studied by researchers and medical professionals, many people are lacking knowledge of the disease, and vaccines and antiviral drugs are still not available to directly prevent or treat the infection. The last major outbreak of SARS-CoV in China was in 2003. It was an acute respiratory infectious ailment with a large risk of demise. China managed the SARS outbreak through numerous inspections and effective preventive measures. The time of incubation for COVID-19 is substantial and very long compared to SARS. Many studies computed different periods of incubation for the COVID-19, as an example, 5.2 days [13], 3.0 days [14], and 4.75 days [15]. In [14], an incubation period of up to 24 days is reported, this extends to 38 days as reported in Enshi Tujia and Miao autonomous prefecture in Hubei province of China. Notice that people afflicted with asymptoms are very numerous [16], and in comparison to SARS-CoV and MERS-CoV the death rate is much inferior [14]. Genetic virus studies show that SARS-CoV and 2019-nCoV are 85% homologous [17], but 2019-nCoV binds ACE2 to an affinity higher than SARS-CoVS [18]. In the positive reported cases induced at COVID-19, SARS will be surpassed by the end of 29 January 2020. The variability of the asymptomatic cases, the incubation time and the super transmissibility of the virus bring considerable difficulties in controlling the epidemics. There is also very recent and novel research that discussed the dynamic and transmission of COVID-19 [19, 20].

Over the last few decades, many scientists have shown that the fractional models can more accurately explain natural phenomena than the differential equations of the integer order. Because of this advantage, the fractional calculus has taken on the importance and popularity of modeling realistic cases, especially those with memory effects [21–23]. Furthermore, applications of the fractional calculus and mathematical modeling are found in many fields of social sciences, engineering, and mathematical biology [24–32]. Given this importance, we feel inspired to investigate and examine a new fractional version of the model involving Caputo operator. To the best of our knowledge, this is the first time Caputo operator has been employed for the model being considered.

## Formulation of the Caputo SIR model

Although there have been various complex models proposed recently for transmission dynamics of COVID-19 as discussed in Sect. 1, we use a relatively simple and most popular model used in the field of mathematical epidemiology. The SIR model [1] is the stepping stone of all the models proposed after it. This is the reason to choose the SIR model to investigate dynamics of the COVID-19 pandemic while using the Caputo differential operator. The SIR model consists of three mutually distinct categories. First are those people who could potentially catch the disease $S(t)$, second are those who currently have the disease and can infect others $I(t)$ (infectives), and then $R(t)$ stands for the removed, and this is the group of people who have already caught the disease and have now either recovered from the disease or have died with the disease. With all mathematical models, we have to make various assumptions to simplify the real world phenomena because things are just too complicated to express everything in a set of simple equations, so the first assumption that we make here is that the epidemic is sufficiently short, so it does not last that long so that we can assume that the total population remains constant. The second assumption in the model relates to the way in which the disease is transmitted, and we assume that the rate of increase in the infectives is proportional to the contact between the susceptibles and the infectives, and we assume that this occurs at a constant rate. Finally, our third assumption relates to the removal rate and this is the category $R(t)$, so there is a constant rate which could be a death rate or a recovery rate. Having made the assumptions, a set of equations (1)–(3) that are going to govern the model can be written down:

1 $$\begin{aligned} &\mathbb{D} \bigl(S(t) \bigr) = \beta \frac{S I}{N}, \end{aligned}$$

2 $$\begin{aligned} &\mathbb{D} \bigl(I(t) \bigr) = \beta \frac{S I}{N} - \gamma I, \end{aligned}$$

3 $$\begin{aligned} &\mathbb{D} \bigl(R(t) \bigr) = \gamma I, \end{aligned}$$

where $N = S+I+R$ and $\mathbb{D}$ stands for the classical integer-order differential operator. We now have three differential equations for three categories of people within the population. So, the number of susceptibles is going to decrease according to the number of contacts (contact rate = *β*) between the infectives and the susceptibles. Similarly, the number of infectives will increase due to contact between people and decrease because of people either recovering or dying as a result of the disease. Finally, the removed category includes people that no longer can catch the disease either because they have recovered or died, and this is going to increase at the constant rate (recovery rate =*γ*) depending on how many infectives there are. Next, we need some initial data before we can solve the system of differential equations. Therefore,

4 $$ S(0) = S_{0} >0,\qquad I(0) = I_{0} > 0,\qquad R(0) = R_{0} \geq 0. $$

There are many instances wherein classical epidemiological models have been re-investigated using operators of nonlocal nature. It is because of their memory property which makes them the most suitable tool to capture dynamics for the spread of a disease since epidemics are known to have memory retaining characteristics.

Having been inspired by a plethora of research works carried out in fractional mathematical epidemiology (see, for example, [33–35] and most of the references cited therein), we have introduced the following Caputo-type SIR model wherein its distinguished feature is that the dimensional inconsistency of the model has been removed by carrying the fractional order *ν* in the power of biological parameters *β* and *γ*:

5 $$ \begin{aligned} &{}^{\mathrm{C}}\mathbb{D}_{0,t}^{\nu } \bigl(S(t) \bigr)= \frac{1}{\Gamma (1-\nu )} \int _{0}^{t} (t-\psi )^{-\nu } \frac{d}{dt} S(\psi )\,d\psi = -\beta ^{\nu }\frac{SI}{N}, \\ &{}^{\mathrm{C}}\mathbb{D}_{0,t}^{\nu } \bigl(I(t) \bigr)= \frac{1}{\Gamma (1-\nu )} \int _{0}^{t} (t-\psi )^{-\nu } \frac{d}{dt}I(\psi )\,d\psi = \beta ^{\nu }\frac{SI}{N} - \gamma ^{\nu } I , \\ &{}^{\mathrm{C}}\mathbb{D}_{0,t}^{\nu } \bigl(R(t) \bigr)= \frac{1}{\Gamma (1-\nu )} \int _{0}^{t} (t-\psi )^{-\nu } \frac{d}{dt}R(\psi )\,d\psi = \gamma ^{\nu } I, \end{aligned} $$

where ${}^{\mathrm{C}}\mathbb{D}_{0,t}^{\nu }$ stands for the Caputo (noninteger-order) differential operator with order of system (5) to be $\nu \in (0,1)$.

## Existence and uniqueness results

Among the vital areas in the concept of noninteger-order differential equations is the concept of existence and uniqueness of solutions in a dynamical system. Recently, the theory has attracted many researchers’ attention [36]. By means of a fixed point theorem, we report the existence and uniqueness of (5). Take into account the proposed model (5) in the form as comes next:

6 $$ \textstyle\begin{cases} {}^{C}\mathcal{D}^{\nu }_{0^{+}}S(t)=\Lambda _{1}(t, S, I, R),\\ {}^{C}\mathcal{D}^{\nu }_{0^{+}}I(t)=\Lambda _{2}(t, S, I, R), \\ {}^{C}\mathcal{D}^{\nu }_{0^{+}}R(t)=\Lambda _{3}(t, S, I, R), \end{cases} $$

with

7 $$ \textstyle\begin{cases} \Lambda _{1}(t, S, I, R)=-\beta ^{\nu }\frac{SE}{N},\\ \Lambda _{2}(t, S, I, R)=-\beta ^{\nu }\frac{SI}{N}-\gamma ^{\nu }I, \\ \Lambda _{3}(t, S, I, R)=\gamma ^{\nu }E. \end{cases} $$

Therefore, (5) can accommodate the form

8 $$ \textstyle\begin{cases} {}^{C}\mathcal{D}^{\alpha }_{0}\Upsilon (t)=\mathcal{R}(t, \Upsilon (t));\quad t\in J=[0, b], 0< \alpha \le 1,\\ \Upsilon (0)=\Upsilon _{0}\ge 0, \end{cases} $$

only if

9 $$ \textstyle\begin{cases} \Upsilon (t)=(S, I, R)^{T},\\ \Upsilon (0)=(S_{0}, I_{0}, R_{0})^{T}, \\ \mathcal{R}(t, \Upsilon (t))=(\Theta _{i}(t, S, I, R))^{T}, \quad i=1,2,3, \end{cases} $$

with *T* representing the transpose. Now, we can write (8) as

10 $$ \begin{aligned} \Upsilon (t)&=\Upsilon _{0}+ \mathcal{J}_{0^{+}}^{\nu } \mathcal{R}\bigl(t, \Upsilon (t)\bigr) \\ &=\Upsilon _{0}+\frac{1}{\Gamma (\nu )} \int _{0}^{t}(t-\kappa )^{\nu -1} \mathcal{R} \bigl(\kappa, \Upsilon (\kappa )\bigr)\,d\kappa. \end{aligned} $$

Consider the Banach space on $[0, b]$, with $[0, b]$ denoting a set of continuous functions from on *R* with the associated norm given by $\|\Upsilon \|=\sup_{t\in \mathcal{J}}|\Upsilon |$ be $\mathbb{E}= C([0, b]; R)$. Also, take into account the following theorem.

### Theorem 3.1

*Surmise that* $\mathcal{R}\in C([\mathcal{J}, \mathbb{R}])$ *and also maps* $\mathcal{J}\times \mathbb{R}^{5}$ (*bounded subset*) *to relatively* $\mathbb{R}$ (*compact subsets*). *Additionally*, ∃ *a constant* $\mathcal{L}_{\mathcal{R}}>0$ *such that* $(\mathit{A}_{1})$ $|\mathcal{R}(t, \Upsilon _{1}(t))-\mathcal{R}(t, \Upsilon _{2}(t))| \le \mathcal{L}_{\mathcal{R}}|\Upsilon _{1}(t)-\Upsilon _{2}(t)|; \forall t\in \mathcal{J}$ *and each* $\Upsilon _{1},\Upsilon _{2}\in C([\mathcal{J}, \mathbb{R}])$. (10) *which is an equivalence of* (5) *accommodates a unique solution only if* $\Omega \mathcal{L}_{\mathcal{R}}<1$, *and*

$$ \Omega =\frac{b^{\nu }}{\Gamma (\nu +1)}. $$

### Proof

Take into account $P:\mathbb{E}\rightarrow \mathbb{E}$ expressed as

11 $$ (P\Upsilon ) (t)=\Upsilon _{0}+\frac{1}{\Gamma (\nu )} \int _{0}^{t}(t- \kappa )^{\nu -1}\mathcal{R} \bigl(\kappa, \Upsilon (\kappa )\bigr)\,d\kappa. $$

(11) shows that the unique solution for (5) represents the fixed point of *P*. Moreover, take $\sup_{t\in \mathcal{J}}\|\mathcal{R}(t, 0)\|=M_{1}$ and $\kappa \ge \|\Upsilon _{0}\|+\Omega M_{1}$. Therefore, it will suffice to verify $P\mathbb{H}_{\kappa }\subset \mathbb{H}_{\kappa }$, and the set given by $\mathbb{H}_{\kappa }=\{\Upsilon \in \mathbb{E}: \|\Upsilon \|\le \kappa \}$ is convex and closed. Now, for any $\Upsilon \in \mathbb{H}_{\kappa }$, we have

12 $$ \begin{aligned} \bigl\vert (P\Upsilon ) (t) \bigr\vert &\le \vert \Upsilon _{0} \vert + \frac{1}{\Gamma (\nu )} \int _{0}^{t}(t-\kappa )^{\nu -1} \bigl\vert \mathcal{R}\bigl( \kappa, \Upsilon (\kappa )\bigr) \bigr\vert \,d\kappa \\ &\le \Upsilon _{0}+\frac{1}{\Gamma (\nu )} \int _{0}^{t}(t-\kappa )^{ \nu -1} \bigl[ \bigl\vert \mathcal{R}\bigl(\kappa, \Upsilon (\kappa )\bigr)-\mathcal{R}( \kappa, 0) \bigr\vert + \bigl\vert \mathcal{R}(\kappa, 0) \bigr\vert \bigr] \,d\kappa \\ &\le \Upsilon _{0}+ \frac{(\mathcal{L}_{\mathcal{R}}\kappa +M_{1})}{\Gamma (\nu )} \int _{0}^{t}(t- \kappa )^{\nu -1}\,d\kappa \\ &\le \Upsilon _{0}+ \frac{(\mathcal{L}_{\mathcal{R}}\kappa +M_{1})}{\Gamma (\nu +1)}b^{ \nu } \\ &\le \Upsilon _{0}+\Omega (\mathcal{L}_{\mathcal{R}}\kappa +M_{1}) \\ &\le \kappa, \end{aligned} $$

which affirms the result. Further, for given $\Upsilon _{1},\Upsilon _{2}\in \mathbb{E}$, one reaches

13 $$ \begin{aligned} \bigl\vert (P\Upsilon _{1}) (t)-(P\Upsilon _{2}) (t) \bigr\vert &\le \frac{1}{\Gamma (\nu )} \int _{0}^{t}(t-\kappa )^{\nu -1} \bigl\vert \mathcal{R}\bigl( \kappa, \Upsilon _{1}(\kappa )\bigr)-\mathcal{R} \bigl(\kappa, \Upsilon _{2}( \kappa )\bigr) \bigr\vert \,d\kappa \\ &\le \frac{\mathcal{L}_{\mathcal{R}}}{\Gamma (\nu )} \int _{0}^{t}(t- \kappa )^{\nu -1} \bigl\vert \Upsilon _{1}(\kappa )-\Upsilon _{2}(\kappa ) \bigr\vert \,d \kappa \\ &\le \Omega \mathcal{L}_{\mathcal{R}} \bigl\vert \Upsilon _{1}(t)- \Upsilon _{2}(t) \bigr\vert , \end{aligned} $$

indicating that $\|(P\Upsilon _{1})-(P\Upsilon _{2})\|\le \Omega \mathcal{L}_{ \mathcal{R}}\| \Upsilon _{1}-\Upsilon _{2}\|$. Thus, as a consequence of the Banach contraction rule, (5) possesses a unique solution on $\mathcal{J}$. □

Now, we want show the existence of solutions of (5) by means of the Schauder fixed point principle.

### Lemma 3.2

*Take* $M\ne \emptyset $ *as a bounded*, *convex*, *and closed subset of a Banach space* $\mathbb{E}$. *Consider* $P_{1}, P_{2}$ *as operators in obedience of the following*:

- $P_{1}\Upsilon _{1}+P_{2}\Upsilon _{2}\in M$, *when* $\Upsilon _{1},\Upsilon _{2}\in M$;
- $P_{1}$ *is continuous and compact*;
- $P_{2}$ *is a contraction mapping*.

*Then* $\exists u\in M$ *so that* $u=P_{1}u+P_{2}u$.

### Theorem 3.3

*Surmise that* $\mathcal{R}:\mathcal{J}\times \mathbb{R}^{5}\rightarrow \mathbb{R}$ *is continuous and satisfies condition* $(A_{1})$. *Additionally*, *take into account that* $(A_{2})$ $|\mathcal{R}(t, \Upsilon )|\le \mathcal{K}(t) for all (t, \Upsilon )\in \mathcal{J}\times \mathbb{R}^{5} and \mathcal{K}\in C([0, b], \mathbb{R}_{+})$.

*Then* (5) *possesses at least one solution on* $\mathcal{J}$ *only if*

$$ \mathcal{L}_{K} \bigl\Vert \Upsilon _{1}(t_{0})- \Upsilon _{2}(t_{0}) \bigr\Vert < 1. $$

### Proof

Consider $\sup_{t\in \mathcal{J}}|\mathcal{K}(t)|=\|\mathcal{K} \|$ and $\zeta \ge \|\Upsilon _{0}\|+\Omega \|\mathcal{K}\|$, with $\mathbf{B}_{\zeta }=\{\Upsilon \in \mathbb{E}: \|\Upsilon \|\le \zeta \}$. Take into account $P_{1}, P_{2}$ on $\mathbf{B}_{\zeta }$ given by

$$ (P_{1}\Upsilon ) (t)=\frac{1}{\Gamma (\nu )} \int _{0}^{t}(t-\kappa )^{ \nu -1}\mathcal{R} \bigl(\kappa, \Upsilon (\kappa )\bigr)\,d\kappa, \quad t\in \mathcal{J}, $$

and

$$ (P_{2}\Upsilon ) (t)=\Upsilon (t_{0}),\quad t\in \mathcal{J}. $$

Therefore, $\forall \Upsilon _{1},\Upsilon _{2}\in \mathbf{B}_{\zeta }$, we have

14 $$ \begin{aligned} \bigl\Vert (P_{1}\Upsilon _{1}) (t)+(P_{2}\Upsilon _{2}) (t) \bigr\Vert &\le \Vert \Upsilon _{0} \Vert +\frac{1}{\Gamma (\nu )} \int _{0}^{t}(t-\kappa )^{ \nu -1} \bigl\Vert \mathcal{R}\bigl(\kappa, \Upsilon _{1}(\kappa )\bigr) \bigr\Vert \,d \kappa \\ &\le \Vert \Upsilon _{0} \Vert +\Omega \Vert \mathcal{K} \Vert \\ &\le \zeta < \infty. \end{aligned} $$

Thus, $P_{1}\Upsilon _{1}+P_{2}\Upsilon _{2}\in \mathbf{B}_{\zeta }$.

Now, the contraction of $P_{2}$ will be proved.

Given any $t\in \mathcal{J}$ and $\Upsilon _{1},\Upsilon _{2}\in \mathbf{B}_{\zeta }$, it yields

15 $$ \bigl\Vert (P_{2}\Upsilon _{1}) (t)-(P_{2} \Upsilon _{2}) (t) \bigr\Vert \le \bigl\Vert \Upsilon _{1}(t_{0})- \Upsilon _{2}(t_{0}) \bigr\Vert . $$

Having $\mathcal{R}$ as a continuous function, $P_{1}$ is continuous. Furthermore, $\forall t\in \mathcal{J}$ and $\Upsilon _{1}\in \mathbf{B}_{\zeta }$,

$$ \Vert P_{1}\Upsilon \Vert \le \Omega \Vert \mathcal{K} \Vert < +\infty, $$

$P_{1}$ is uniformly bounded. At last, we prove that $P_{1}$ is compact. Start with $\sup_{(t, \Upsilon )\in \mathcal{J}\times \mathbf{B}_{ \zeta }}|\mathcal{R}(\kappa, \Upsilon (\kappa ))|=\mathcal{R}^{*}$, which yields

16 $$ \begin{aligned} \bigl\vert (P_{1}\Upsilon ) (t_{2})-(P_{1}\Upsilon ) (t_{2}) \bigr\vert ={}& \frac{1}{\Gamma (\nu )} \biggl\vert \int _{0}^{t_{1}}\bigl[(t_{2}-\kappa )^{\nu -1}-(t_{1}- \kappa )^{\nu -1}\bigr]\mathcal{R} \bigl(\kappa, \Upsilon (\kappa )\bigr)\,d\kappa \\ &{}+ \int _{t_{1}}^{t_{2}}(t_{2}-\kappa )^{\nu -1}\mathcal{R}\bigl(\kappa, \Upsilon (\kappa )\bigr)\,d\kappa \biggr\vert \\ \le{}& \frac{\mathcal{R}^{*}}{\Gamma (\nu )} \bigl[2(t_{2}-t_{1})^{\nu }+ \bigl(t_{2}^{ \nu }-t_{1}^{\nu }\bigr) \bigr] \\ \rightarrow{}& 0,\quad \text{as }t_{2}\rightarrow t_{1}. \end{aligned} $$

Hence, $P_{1}$ is equicontinuous and so is relatively compact on $\mathbf{B}_{\zeta }$. In accordance with the Arzelá–Ascoli principle, $P_{1}$ is compact on $\mathbf{B}_{\zeta }$. Having satisfied all the claims of Lemma 3.2, (5) possesses at least one solution on $\mathcal{J}$. □

## Stability analysis

Here, the stability of (5) will scrutinize the aspect of Ulam–Hyers and generalized Ulam–Hyers [37, 38]. It has been proved that stability analysis is important for an approximate solution. Assume $\varepsilon >0$ and take into account the inequality

17 $$ \bigl\vert {}^{C}\mathcal{D}_{0^{+}}^{\nu } \bar{\Upsilon }(t)-\mathcal{R}\bigl(t, \bar{\Upsilon }(t)\bigr) \bigr\vert \leq \varepsilon, \quad t\in J, $$

and $\varepsilon =\max (\varepsilon _{j})^{T}, j=1,\ldots 5$.

### Definition 1

(8), which is the equivalence of (5), is Ulam–Hyers stable if ∃ $\mathcal{X}_{\mathcal{R}}>0$, so that ∀ $\varepsilon >0$ and a solution $\bar{\Upsilon }\in \mathbb{E}$ holds for (1), there is the unique solution $\Upsilon \in \mathbb{E}$ for (8), with

$$ \bigl\vert \bar{\Upsilon }(t)-\Upsilon (t) \bigr\vert \leq \mathcal{X}_{\mathcal{R}} \varepsilon,\quad t\in J, $$

where $\mathcal{X}_{\mathcal{R}}=\max (\mathcal{X}_{\mathcal{R}_{j}})^{T}$.

### Definition 2

(8), which is the equivalence of (5), is referred to as generalized Ulam–Hyers stable if ∃ a continuous function $\vartheta _{\mathcal{R}}: \mathbb{R}_{+} \rightarrow \mathbb{R}_{+}$, with $\vartheta _{\mathcal{R}}(0)=0$, so that ∀ solution $\bar{\Upsilon }\in \mathbb{E}$ of (17), there is the unique solution $\Upsilon \in \mathbb{E}$ for (8), so that

$$ \bigl\vert \bar{\Upsilon }(t)-\Upsilon (t) \bigr\vert \leq \vartheta _{\mathcal{R}} \varepsilon,\quad t\in J, $$

where $\vartheta _{\mathcal{R}}=\max (\vartheta _{\mathcal{R}_{j}})^{T}$.

### Remark 1

A function $\bar{\Upsilon }\in \mathbb{E}$ satisfies (17) if and only if ∃ a function $h\in \mathbb{E}$ with the property as comes next:

(i) $|h(t)|\leq \varepsilon, h=\max (h_{j})^{T}, t\in J$;
(ii) ${}^{C}\mathcal{D}_{0^{+}}^{\nu }\bar{\Upsilon }(t)=\mathcal{R}(t, \bar{\Upsilon }(t))+h(t), t\in J$.

### Lemma 4.1

*Surmise that* $\bar{\Upsilon }\in \mathbb{E}$ *holds for* (17), *then* ϒ̄ *holds for the following*:

18 $$ \biggl\vert \bar{\Upsilon }(t)-\bar{\Upsilon }_{0}- \frac{1}{\Gamma (\nu )} \int _{0}^{t}(t-\kappa )^{\nu -1}\mathcal{R} \bigl(\kappa, \bar{\Upsilon }( \kappa )\bigr)\,d\kappa \biggr\vert \leq \Omega \varepsilon. $$

### Proof

By utilizing (ii) of 1,

$$ ^{C}\mathcal{D}_{0^{+}}^{\nu }\bar{\Upsilon }(t)= \mathcal{R}\bigl(t, \bar{\Upsilon }(t)\bigr)+h(t) $$

and Lemma 4.1, we have that

19 $$ \bar{\Upsilon }(t)=\bar{\Upsilon }_{0}+\frac{1}{\Gamma (\nu )} \int _{0}^{t}(t- \kappa )^{\nu -1}\mathcal{R} \bigl(\kappa, \bar{\Upsilon }(\kappa )\bigr)\,d \kappa +\frac{1}{\Gamma (\nu )} \int _{0}^{t}(t-\kappa )^{\nu -1}h( \kappa ) \,d\kappa. $$

Considering (i) of 1 yields

20 $$ \begin{aligned} \biggl\vert \bar{\Upsilon }(t)-\bar{\Upsilon }_{0}- \frac{1}{\Gamma (\nu )} \int _{0}^{t}(t-\kappa )^{\nu -1}\mathcal{R} \bigl( \kappa, \bar{\Upsilon }(\kappa )\bigr)\,d\kappa \biggr\vert &\le \frac{1}{\Gamma (\nu )} \int _{0}^{t}(t-\kappa )^{\nu -1} \bigl\vert h(\kappa ) \bigr\vert \,d \kappa \\ &\leq \Omega \varepsilon. \end{aligned} $$

Hence, we complete the result. □

### Theorem 4.2

*Surmise that* $\mathcal{R}: J\times \mathbb{R}^{5}\rightarrow \mathbb{R}$ *is continuous* ∀ $\Upsilon \in \mathbb{E}$ *and assumption* $(\mathit{A}_{1})$ *holds with* $1-\Omega \mathcal{L}_{\mathcal{R}}>0$. *Then* (8), *which is the equivalence of* (5), *is Ulam–Hyers and*, *consequently*, *generalized Ulam–Hyers stable*.

### Proof

Surmise that $\bar{\Upsilon }\in \mathbb{E}$ holds for (17) and $\Upsilon \in \mathbb{E}$ is a unique solution of (8). Therefore, ∀ $\varepsilon >0$, $t\in J$ and Lemma 4.1, it yields

$$ \begin{aligned} \bigl\vert \bar{\Upsilon }(t)-\Upsilon (t) \bigr\vert ={}& \max_{t \in \mathcal{J}} \biggl\vert \bar{\Upsilon }(t)-\Upsilon _{0}- \frac{1}{\Gamma (\nu )} \int _{0}^{t}(t-\kappa )^{\nu -1}\mathcal{R} \bigl( \kappa, \Upsilon (\kappa )\bigr)\,d\kappa \biggr\vert \\ \le {}&\max_{t\in \mathcal{J}} \biggl\vert \bar{\Upsilon }(t)- \bar{ \Upsilon }_{0}-\frac{1}{\Gamma (\nu )} \int _{0}^{t}(t-\kappa )^{ \nu -1}\mathcal{R} \bigl(\kappa, \bar{\Upsilon }(\kappa )\bigr)\,d\kappa \biggr\vert \\ &{}+ \max_{t\in \mathcal{J}}\frac{1}{\Gamma (\nu )} \int _{0}^{t}(t- \kappa )^{\nu -1} \bigl\vert \mathcal{R}\bigl(\kappa, \bar{\Upsilon }(\kappa )\bigr)- \mathcal{R}\bigl( \kappa, \Upsilon (\kappa )\bigr) \bigr\vert \,d\kappa \\ \leq{}& \biggl\vert \Upsilon (t)-\bar{\Upsilon }_{0}- \frac{1}{\Gamma (\nu )} \int _{0}^{t}(t-\kappa )^{\nu -1}\mathcal{R} \bigl(\kappa, \bar{\Upsilon }( \kappa )\bigr)\,d\kappa \biggr\vert \\ &{}+\frac{\mathcal{L}_{\mathcal{R}}}{\Gamma (\nu )} \int _{0}^{t}(t- \kappa )^{\nu -1} \bigl\vert \bar{\Upsilon }(\kappa )-\Upsilon (\kappa ) \bigr\vert \,d\kappa \\ \leq{}& \Omega \varepsilon +\Omega \mathcal{L}_{\mathcal{R}} \bigl\vert \bar{ \Upsilon }(t)-\Upsilon (t) \bigr\vert . \end{aligned} $$

So,

$$ \Vert \bar{\Upsilon }-\Upsilon \Vert \leq \mathcal{X}_{\mathcal{R}} \varepsilon, $$

where

$$ \mathcal{X}_{\mathcal{R}}= \frac{\Omega }{1-\Omega \mathcal{L}_{\mathcal{R}}}. $$

Equating $\vartheta _{\mathcal{R}}(\varepsilon )=\mathcal{X}_{\mathcal{R}} \varepsilon $ so that $\vartheta _{\mathcal{R}}(0)=0$, one concludes that (5) is stable for both Ulam–Hyers and generalized Ulam–Hyers. □

## Estimation of parameters

A disease model like the one being investigated in the present research study is widely accepted and validated if its simulations for infectious compartment agree well with those of available real cases of the disease. In order to do so, one needs to obtain values of the parameters of the model in a best possible way so that they cause the model to be in good agreement with the available data. This brings into attention a few existing methods for getting best values of such parameters. The Bayesian technique, probability plotting, maximum likelihood estimation, and least-squares method are among the methods that exist for parameter estimation. In this study, we have used the least-squares method to compute the parameters (*β*= transmission rate and *γ*= recovery rate) of model (5). Since there are only two non-demographical parameters for the proposed model, they are best fitted using the real cases of COVID-19 pandemic throughout Pakistan (source http://covid.gov.pk/stats/pakistan). Daily cases of the disease are taken from 1 April to 15 May 2020 when the present research study was being conceived and prepared.

In the least-squares approach, the objective function requires to be minimized. The objective is based upon adjusting the parameters for a model function to fit a data set in a best possible way. A simple data set consists of *n* points (data pairs) $(x_{k},y_{k}),k=1,\ldots,n$, where $x_{k}$ shows an independent variable and $y_{k}$ stands for a dependent variable whose value is computed using observation. The model function possesses the form $g(x,p)$, where *m* adjustable parameters are kept in the vector *p*. The goal is to notice the parameters for the model that fit the data in a best possible way. Such a fit of a model to a data point is measured by its residual, defined as the difference between the real available value of the dependent variable and the value predicted by the model:

21 $$ r_{k}=y_{k} - g(x_{k},p). $$

The least-squares method obtains the optimal parameter values by minimizing the sum of squared residuals as shown below:

22 $$ S=\sum_{k=1}^{n} (r_{k})^{2}= \sum_{k=1}^{n} \bigl(y_{k} - g(x_{k},p)\bigr)^{2}. $$

**Figure 1:**
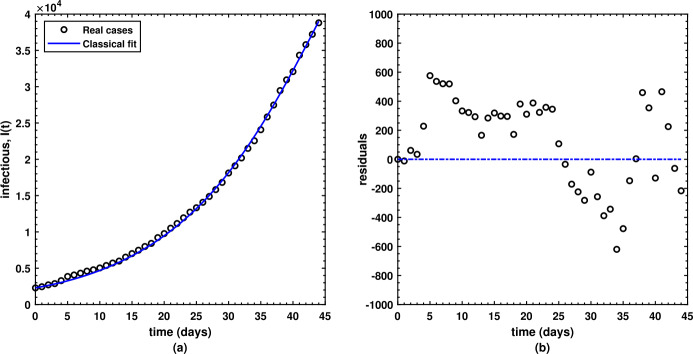
(a) Best data fitting for COVID-19 daily cases (black circles) in Pakistan from 1 April to 15 May 2020 with the infectious class $I(t)$ (solid blue line) of the model under the classical case ($\nu = 1$) and the (b) respective residuals

**Figure 2:**
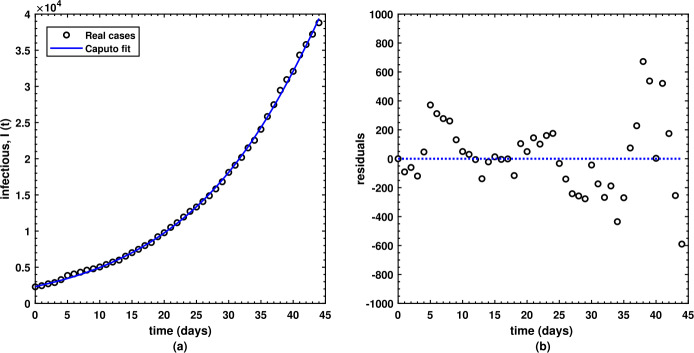
(a) Best data fitting for COVID-19 daily cases (black circles) in Pakistan from 1 April to 15 May 2020 with the infectious class $I(t)$ (solid blue line) of the model under the Caputo operator ($\nu = 8.561591\text{e-}01$) and the (b) respective residuals

For initial conditions, the overall population of Pakistan is found to be $N(0)=212.2$M, the initial susceptible population is estimated to be $S(0)=212,197,711$, the initial exposed population is estimated to be $E(0)=2,289$, and the initial recovered population is estimated to be $R(0)=0$. There are only two biological parameters best fitted via the least-squares fitting method thereby yielded best fit of the model’s solution to the real cases chosen from Pakistan as depicted by Fig. 1 under the classical situation, that is, when $\nu = 1$ wherein the best parameters are as follows: $\beta = 3.0918$ and $\gamma =3.0190$ for the transmission and recovery rate, respectively. The average absolute relative error between the real cases and the model’s simulations for the infectious compartment is decreased. Such a value for the error is approximately $3.3603e-02$. Figure 1 shows the real cases by black circles, while the best fitted curve is shown by the blue solid line and the residuals are shown by side. The biological parameters included in the model are listed in Table 1 along with their best estimated values obtained via the least-squares technique. These parameters have finally produced the value of the basic reproduction number equal to ${\mathcal{R}}_{0}=1.0241$ for the real COVID-19 cases under the classical situation in Pakistan from 1 April to 15 May 2020. Moreover, similar analysis for the Caputo fractional-order model is carried out wherein the best fitted parameters are found as $\beta = 3.9405$ and $\gamma =3.8010$ for the transmission and recovery rate, respectively. One distinguished feature in the present research study is the computation of an optimum value of the fractional-order parameter *ν* which is 8.561591e-01. Hence, the basic reproduction number is obtained as ${\mathcal{R}}_{0}= 1.0313$ and the corresponding average absolute relative error is 1.7360e-02 for the Caputo model, whereas 3.3603e-02 is the average absolute relative error when $\nu = 1$. This clearly reveals the superiority of the fractional operator over the classical one. Figure 2 shows the real cases by black circles, while the best fitted curve obtained under the Caputo fractional case is shown by the blue solid line and the residuals are shown by side.

**Table 1:** Best fitted parameters employed in model (5) for its classical $\nu = 1$ and fractional $\nu = 8.561591\text{e-}01$ version

Parameter	Description	Value	Sources
*β*	Transmission rate	3.0918^C^ 3.9405^F^	Fitted
*γ*	Recovery rate	3.0190^C^ 3.8010^F^	Fitted

## Sensitivity analysis

In this portion, the concept of sensitivity analysis is employed to discover the robust significance of the generic parameters that are present in the basic reproduction number ${\mathcal{R}}_{0}$. Further, with the aid of parameter values from reliable assumptions, both analytic and numerical values of the parameters in ${\mathcal{R}}_{0}$ are obtained. The analytic expressions obtained can be used to shed some light on how to control the onset of the model in variant localities if and only if the dynamics follow model (1). The threshold value ${\mathcal{R}}_{0}$ is a quantity of which lowering the number to less than unity is considered as the major way of curtailing and aborting the spread of the ailment. The sensitivity index technique is used to measure the most sensitive parameters in the model, those with positive sign are considered as highly and proportionally sensitive for increasing the value of ${\mathcal{R}}_{0}$, while those with negative sign are less sensitive for the decrease of ${\mathcal{R}}_{0}$ and the other category are neutrally sensitive (with zero relative sensitivity). It is popularly known that the cause of raping transmission is associated directly with the basic reproduction number ${\mathcal{R}}_{0}$. The elasticity indices of ${\mathcal{R}}_{0}$ to the associated parameters in the model are defined as follows [39]:

23 $$ \Upsilon ^{{\mathcal{R}}_{0}}_{\mathbf{P_{i}}}= \frac{\partial {\mathcal{R}}_{0}}{\partial \mathbf{P}_{i}}\times \frac{\mathbf{P}_{i}}{{\mathcal{R}}_{0}}, $$

where ${\mathcal{R}}_{0}$ denotes the basic reproduction ratio and $\mathbf{P}_{i}$ is as stated above. ${\mathcal{R}}_{0}$ for model (5) under consideration is defined by the following expression:

24 $$ {\mathcal{R}}_{0}= \biggl(\frac{\beta }{\gamma } \biggr)^{\nu }. $$

Thus, after some computation we reach

25 $$ \begin{aligned} &\Upsilon _{\beta }=\nu, \\ &\Upsilon _{\gamma }=-\nu. \end{aligned} $$

**Figure 3:**
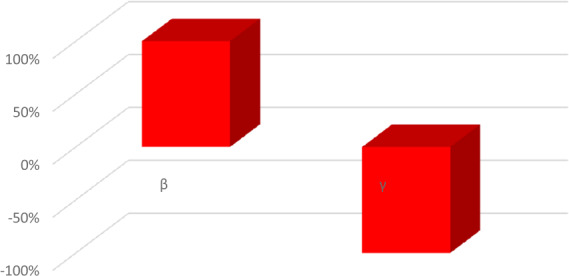
Normalized local sensitivity indices of ${\mathcal{R}}_{0}$ via bar chart

**Figure 4:**
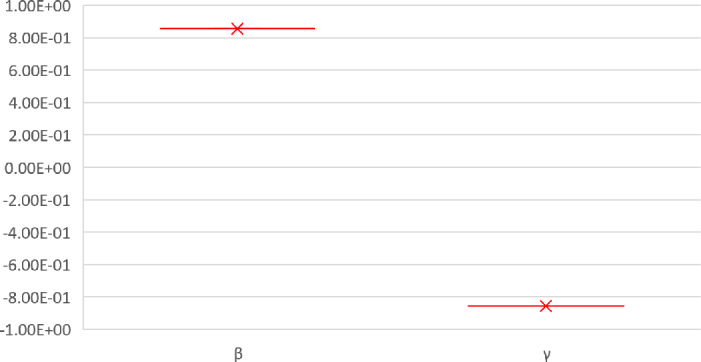
Normalized local sensitivity indices of ${\mathcal{R}}_{0}$ for each parameter

The numerical values showing the relative significance of the $R_{0}$ parameters are given in Table 2. Positive relationship parameter is *β*, while that of negative relations is *γ*. A negative relationship indicates that an increase in this parameter’s value would help to reduce the brutality of the disease. While a positive relationship indicates that an increase in the values of that parameter would have a substantial effect on the frequency of the spread of the ailment. Figure 3 falls in a positive upper part, while Fig. 4 falls in a negative lower part as stated in Table 2.

**Table 2:** The elasticity indices for ${\mathcal{R}}_{0}=1.0313$ to the parameters of model (5)

Parameter	Baseline value	Elasticity index
*β*	3.9405^F^	8.561591e-01
*γ*	3.8010^F^	−8.561591e-01

## Numerical simulations

**Figure 5:**
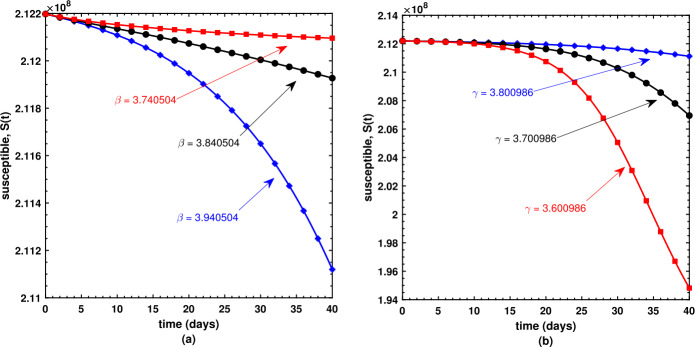
Behavior of the susceptible population under varying values of *β* and *γ*

**Figure 6:**
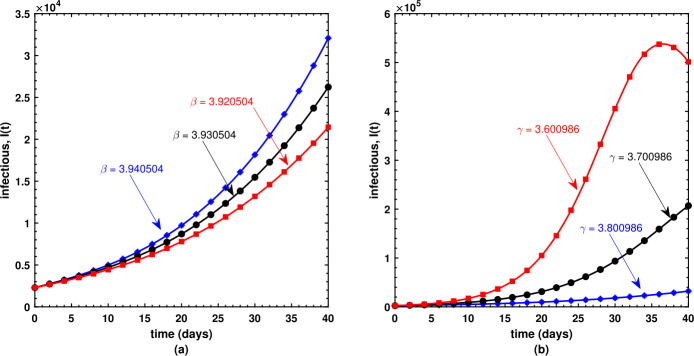
Behavior of the infectious population under varying values of *β* and *γ*

**Figure 7:**
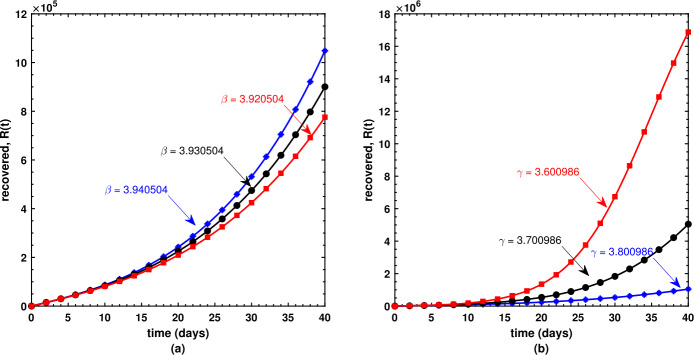
Behavior of the recovered population under varying values of *β* and *γ*

This is the place where we get deep insights into the model’s dynamical behavior. The present section offers the numerical simulations of Caputo model (5) while using the biological parameters as listed in Table 1. We have employed the FDE12 technique in order to get the solution of the nonlinear model shown in equations (5) and achieved the graphical results based upon parameters which are taken to be variables. Susceptible $S(t)$, infectious $I(t)$, and recovered $R(t)$ populations are investigated with different values of the parameters. It may also be noted that the optimized value of the fractional order $\nu = 8.561591\text{e-}01$ is used during each simulation. As shown in (a) plot of Fig. 5, the susceptible individuals decrease as the transmission rate increases; in contrast, the susceptible individuals decrease as the recovery rate decreases as shown by (b) plot of Fig. 5. Similarly, the slightly increased value of the transmission rate *β* is responsible for the spread of the virus (Fig. 6(a)), and the number of infectious individuals decreases when there is an improvement in their recovery which is observed in (b) plot of Fig. 6. Surprisingly, an increasing value of *β* causes an increase in the recovered population after a certain level of time interval as shown in (a) plot of Fig. 7, and similar surprising behavior is noted in (b) plot of Fig. 7. However, this should be obvious since with rapid transmission rate there will be more effective strategies used by public health sectors and concerned government to overcome the situation leading to an increase in the recovered population with similar sort of explanation for (b) plot of Fig. 7.

**Figure 8:**
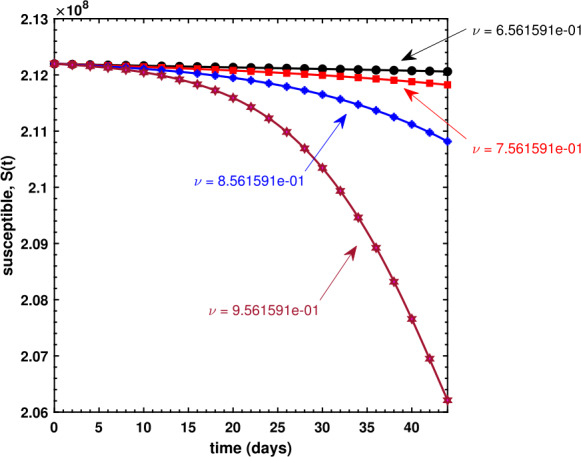
Behavior of the susceptible population for different values of the fractional-order parameter *ν*

**Figure 9:**
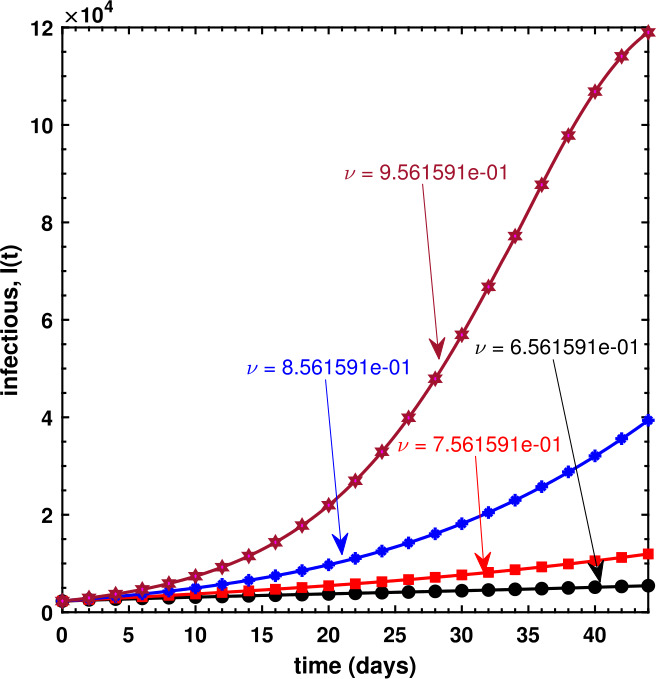
Behavior of the infectious population for different values of the fractional-order parameter *ν*

**Figure 10:**
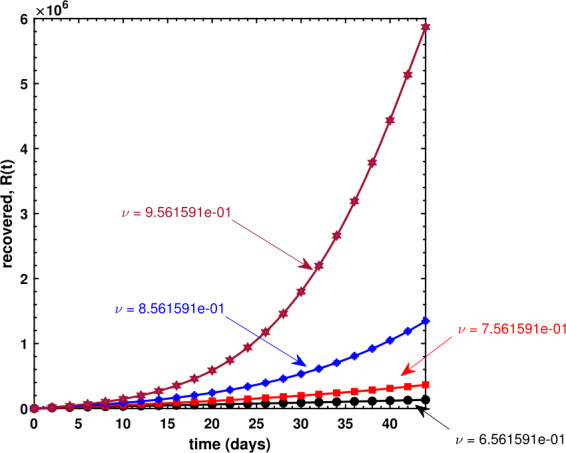
Behavior of the recovered population for different values of the fractional-order parameter *ν*

**Figure 11:**
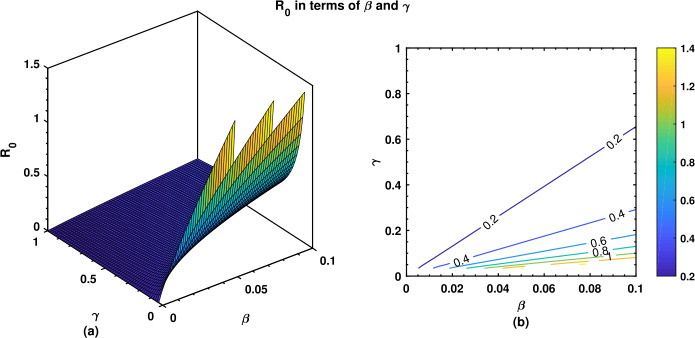
Behavior of the basic reproductive number

We have also investigated the effects of fractional order *ν* on the dynamical behavior of Caputo model (5) for all three compartments. As can be seen in Fig. 8, the susceptible population decreases if *ν* increases, whereas Fig. 9 shows an increase in the infectious population for increasing values of *ν*, and this makes sense because the susceptible population, after being infected, is shifted to *I* compartment thereby leading to an increase therein. It is observed in Fig. 10 that the recovered population propels forward if *ν* gets large. Finally, we have also shown the dynamics of the basic reproduction number ${\mathcal{R}}_{0}$ under different effects of the transmission rate *β* and the recovery rate *γ* in Fig. 11, wherein even a slightly larger value of *β* brings the reproduction number near to 1, which is clearly an alarming situation for policy makers to devise an effective approach to prevent ${\mathcal{R}}_{0}$ to be greater than 1.

## Conclusion

Using Caputo differential operator famous to have nonlocal nature, which is perfectly suitable to investigate transmission dynamics of a disease, we have fractionalized the SIR epidemic model having order *ν* without violating dimensional consistency between parameters and the operator itself. The proposed Caputo SIR model is used to comprehend the behavior of the devastating disease of COVID-19 that has recently emerged in the entire community of humankind. The model has shown promising results for it is proved to have a unique solution on the basis of the Banach contraction principle. Using Ulam–Hyers and its generalized version, the Caputo model is shown to be stable. Fitted parameters of the model, using real incidence cases of the virus from 1 April to 15 May 2020, are obtained under the least-squares approach wherein the fractional order *ν* is also optimized to be $8.561591\text{e-}01$: one of the major contributions in the present work. Using these values, it has been proved that the Caputo model outperforms its classical version by 48% while the basic reproductive number is ${\mathcal{R}}_{0}=1.0313$. Sensitivity of the parameters *β* and *γ* relating with ${\mathcal{R}}_{0}$ is also investigated. Various numerical simulations carried out suggest that the epidemic can effectively be controlled only if its contact rate is lowered, and this is possible when people observe social distancing and wear protective masks. On the other hand, an underdeveloped country like Pakistan will be in huge trouble if such measures are not strictly followed upon. Our future study will explore the effects of nonsingular differential operators on the standard SIR model.

## Data Availability

The data used in this study is taken from the Government of Pakistan website for COVID-19 (http://covid.gov.pk/stats/pakistan).
